# Fiberoptic Intubation vs. Video-Assisted Fiberoptic Intubation in a High-Fidelity Pediatric Simulator: A Randomized Controlled Trial

**DOI:** 10.7759/cureus.39280

**Published:** 2023-05-20

**Authors:** Hatsuo Isogai, Taiki Kojima, Hiromi Kako

**Affiliations:** 1 Department of Anaesthesiology, Aichi Children's Health and Medical Center, Obu, JPN; 2 Division of Comprehensive Paediatric Medicine, Nagoya University Graduate School of Medicine, Nagoya, JPN

**Keywords:** perioperative respiratory adverse events, high-fidelity simulator, pediatric anesthesia, video-assisted fiberoptic intubation, fiberoptic intubation, airway management

## Abstract

Introduction: Life-threatening hypoxemia during tracheal intubation is more likely to occur in children than adults due to its unique physiological and anatomical nature. Fiberoptic intubation is widely performed in children with difficult airways. However, mastery of fiberoptic intubation requires substantial training, and novice trainees need to attempt fiberoptic intubation in children at high risk of respiratory-related adverse events. Therefore, a safer method than traditional fiberoptic intubation for children with difficult airways is desirable for novice anesthesia trainees. This study aimed to compare the efficacy of video-assisted fiberoptic intubation (VAFI) with that of traditional fiberoptic intubation (FOI) in a high-fidelity pediatric simulator by medical professionals with no experience in tracheal intubation.

Method: This randomized, controlled, simulation-based study was conducted in a tertiary-care pediatric hospital. Registered nurses working in the operating room were enrolled in this study and randomly assigned to either the FOI or VAFI groups. Participants in the FOI group performed fiberoptic intubation without the aid of any device, whereas those in the VAFI group used a video laryngoscope to obtain a better glottic view. The primary outcome was the time from the moment the tip of the flexible bronchoscope passed between the upper and lower incisors until the completion of tracheal intubation.

Results: A total of 28 participants were enrolled in this study. There was no significant difference in the time until the completion of tracheal intubation between FOI and VAFI, with a median time of 55.0 seconds for FOI and 42.5 seconds for VAFI (P = 0.22). Secondary outcomes, including time until passing the vocal cord, the number of intubation attempts, and the first success rate, did not also illustrate the significant difference between the groups.

Conclusion: This study did not demonstrate the superiority of VAFI over conventional FOI in a high-fidelity pediatric simulator by medical providers with no experience in tracheal intubation.

## Introduction

Minimizing the time required to secure an airway in children during general anesthesia is vital to avoiding critical hypoxemia. In children, high oxygen consumption and low functional residual capacity can lead to significant respiratory-related adverse events [1, 2]. A previous study has shown that longer intubation increases the risk of hypoxemia [3]. In addition, infants have a higher incidence of difficult intubation due to their unique anatomy, including a small oral space, a large tongue, and prominent epiglottis and arytenoids [4]. Therefore, video laryngoscopy can be a reliable choice for the first intubation attempt, as it has a high first-attempt success rate [5].

However, anesthesiologists can encounter situations in which fiberoptic intubation (FOI) is performed instead of video laryngoscopy in children with difficult airways. Visualizing the larynx with a video laryngoscope can be challenging for these children because of poor mouth opening, mandibular hypoplasia, and syndromes linked to facial symmetry. In situations where the screen of a video laryngoscope detects the glottis, it may still be difficult to guide the tip of the tracheal tube towards the glottis due to insufficient angulation [6]. Therefore, anesthesiologists must be familiar with FOI in children. In contrast, mastery of FOI through the clinical experience of tracheal intubation in pediatric patients with difficult airways requires substantial clinical training in actual patients. In addition, repeated attempts at tracheal intubation are likely to cause adverse events. Thus, establishing safe methods to perform FOI in children, considering training situations for novice anesthesia providers, is desired.

Previous studies have compared video laryngoscopy with video-assisted fiberoptic intubation (VAFI) in adults with difficult airways [7, 8]. However, these studies focused on evaluating the most effective intubation technique among all methods. None of these studies identified an optimal approach within the subset of tracheal intubation using a flexible bronchoscope. To the best of our knowledge, no reports have evaluated the VAFI's efficacy in children. Developing a more effective and safer method of intubation using a flexible bronchoscope for novice anesthesia trainees in children has significant clinical implications.

This study aimed to compare the effectiveness of VAFI with FOI in a pediatric high-fidelity simulator performed by medical professionals without tracheal intubation experience. We hypothesized that VAFI would shorten tracheal intubation time compared to simple FOI for healthcare professionals without tracheal intubation experience.

## Materials and methods

Study design, setting, and ethical considerations

This open-label, randomized controlled trial was conducted in a tertiary-care children’s hospital after obtaining approval from the institutional review board at Aichi Children’s Health and Medical Center, Obu, Japan (approval number 2022068, December 28th, 2022). This study was conducted in accordance with the Declaration of Helsinki. 

Inclusion criteria

We enrolled registered nurses working in the investigator’s hospital operating room with no tracheal intubation experience.

Exclusion criteria

We excluded participants who refused to participate in the study.

Randomization and masking

**Figure 1:**
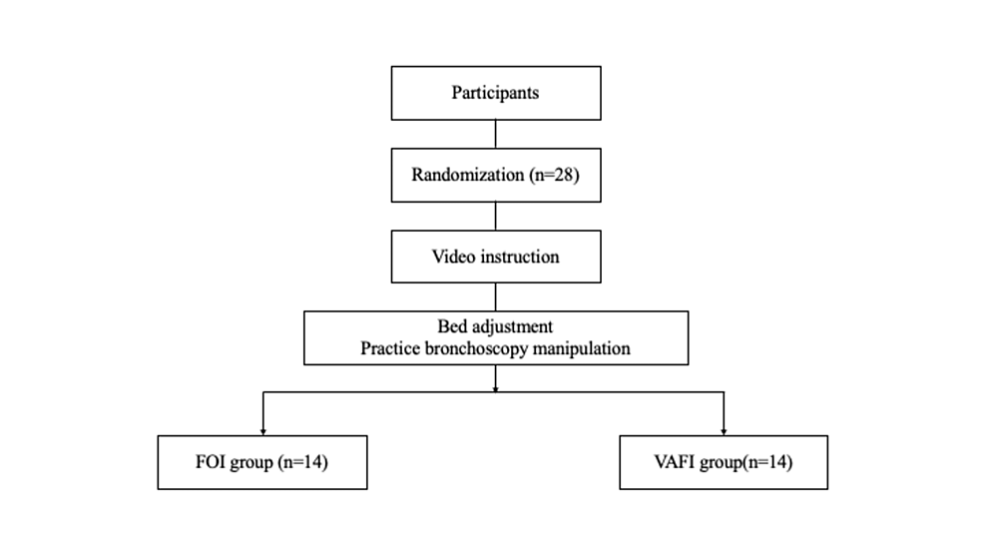
The flow diagram in the trial compares fiberoptic intubation vs. video-assisted fiberoptic intubation.

The study participants were randomly allocated to the VAFI or FOI group (simple randomization). Following the sample size estimations, the research investigator created a random allocation table (1:1 ratio) using Microsoft Excel 2019 (Microsoft, Redmond, WA). The research investigator consecutively allocated the participants to two groups (VAFI or FOI) using a random allocation table (Figure 1). Random allocation was not disclosed to the data analyst until the data analysis was completed.

Instructions for FOI

**Figure 2:**
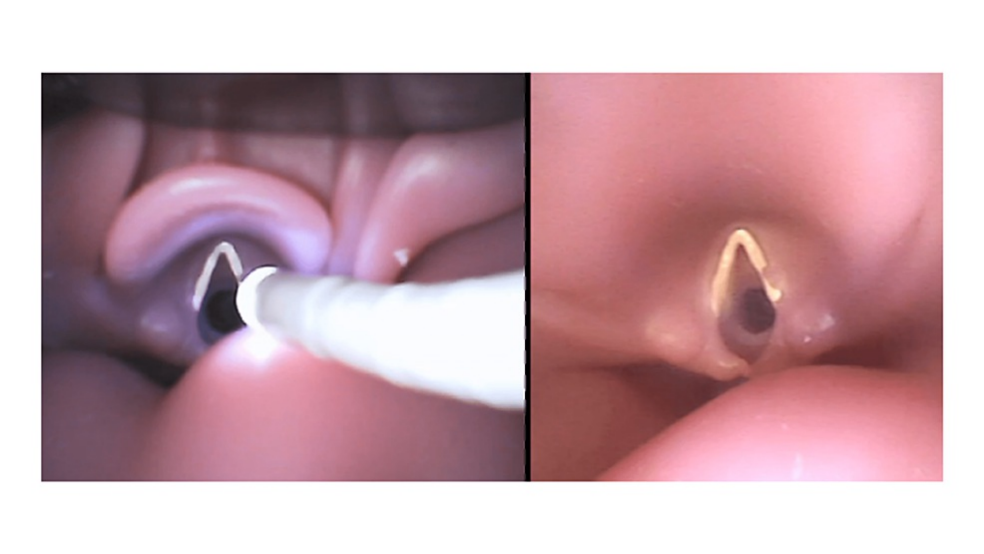
Both the flexible bronchoscope and video laryngoscopy views are displayed side by side on the monitor.

Each participant watched a short instructional video clip (approximately five minutes). The video clip illustrated the anatomy involved in intubation, how to use a flexible bronchoscope (GLIDOSCOPE Bflex2.8; AMCO, Tokyo, Japan), and how to intubate using a bronchoscope (FOI) or the bronchoscope in combination with a disposable video laryngoscope (VAFI) (GLIDESCOPE Spectrum LoPro S2.5; AMCO). For the VAFI procedure, the participants were instructed to watch a monitor reflecting a view from the video laryngoscope but not the view from the flexible bronchoscope (Figure 2).

FOI procedure

The participants had a few minutes to practice basic bronchoscope manipulation before performing the assigned procedure. The operating table’s height was adjusted such that the tip of the flexible bronchoscope was close to the simulator’s mouth when the participant held the flexible bronchoscope in a comfortable position for manipulation.

In both groups, intubation was performed using an endotracheal tube (Microcuff pediatric endotracheal tube, inner diameter 4.0 mm; Avanos Medical Japan, Tokyo, Japan).

In the FOI group, tracheal intubation was initiated by inserting a flexible bronchoscope into the mouth of the simulator. The flexible bronchoscope was advanced over the vocal cord and towards the carina; consequently, a pre-assigned investigator advanced the endotracheal tube into the trachea. Tracheal intubation was confirmed when a bronchoscope visualized the endotracheal tube in the trachea.

In the VAFI group, a pediatric anesthesiologist with more than five years of dedicated experience in pediatric anesthesia performed a video laryngoscopy using a disposable video laryngoscope (GLIDESCOPE Spectrum LoPro S2.5, AMCO). The tip of the laryngoscope video blade was placed in the epiglottic vallecula, resulting in a percentile of glottic opening (POGO) score of 100% for all participants. The flexible bronchoscopic and video laryngoscopic views were displayed side-by-side on a monitor (Figure 2). Upon establishing the laryngoscopic view, the participants started the intubation technique. The intubation process from this point forward was the same as that in the FOI group.

Success and failure of FOI

The first success of tracheal intubation was defined as the confirmation of tracheal tube placement in the trachea by a flexible bronchoscope within 100 seconds after initiating the procedure without inserting the flexible bronchoscope into the esophagus or removing the tip of the flexible bronchoscope from the simulator’s mouth after the initiation of the tracheal intubation technique.

Failure of the first attempt at tracheal intubation was defined as meeting any of the following conditions: It took more than 100 seconds to complete the tracheal intubation procedure (until tracheal tube placement in the trachea was confirmed); the insertion of the flexible bronchoscope into the esophagus or the tip of the flexible bronchoscope was removed from the simulator mouth after the initiation of each procedure.

The number of intubation attempts was quantified. Successful intubation without failure was recorded as one attempt. Removing the flexible bronchoscope from the mouth or inserting it into the esophagus was considered an additional attempt. For example, if a participant inserted the flexible bronchoscope into the esophagus, pulled it back, and inserted it into the trachea, two intubation attempts were recorded.

Data collection 

Primary Endpoint

The primary endpoint was the time interval between the tip of the flexible bronchoscope passing between the upper and lower incisors and the flexible bronchoscope, confirming that an endotracheal tube was placed in the trachea.

Secondary Endpoints

The following secondary endpoints were predetermined before initiating the data collection: the time interval between the tip of the flexible bronchoscope passing between the upper and lower incisors and passing through the vocal cord; the number of intubation attempts; and the first-success rate.

Participants’ information 

This study collected data on participants’ age, sex, and career length as nurses.

Data recording

A research investigator recorded the duration and number of intubations as well as the number of esophageal insertions. Another research investigator performed a video laryngoscopy and advanced the endotracheal tube, sitting next to the participant. They were not supposed to provide any support for the participants, including linguistic advice, throughout the procedures, except for advancing the tracheal tube once the bronchoscope was determined to be inside the trachea.

Data storage and process

The data analyst, unaware of the random allocation results, was responsible for analyzing the data. All collected data were stored on an institutional computer without external access to avoid unexpected data leakage.

Statistical analysis

For summary statistics, categorical variables were described as numbers and percentages, whereas normally and non-normally distributed continuous variables were described as mean and standard deviation or median and interquartile range (IQR). For continuous variables, the significance of differences was determined using the Student’s t-test or the Mann-Whitney U-test based on the distribution. Fisher’s exact or Pearson’s chi-square test was used for appropriate evaluations of categorical variables. Data were analyzed using STATA 17.0 (StataCorp, College Station, TX, USA), with a two-sided p-value <0.05 as the criterion for assessing the null hypothesis for each analysis. The sample size was estimated using G*power (Heinrich Heine University, Düsseldorf, Germany). Secondary endpoints were not assumed to reveal the primary study hypothesis. Thus, we did not draw the primary conclusion from the results of the secondary endpoints to avoid inflation of type I error.

Sample size estimation

We conducted a preliminary randomized study with 12 participants for sample size estimation. All participants were assistant nurses and medical engineers who had never performed FOI. We collected the data using the same methods described earlier. According to the result, the median for the primary outcome was 97.83 seconds (FOI group) and 35.84 seconds (VAFI group), respectively. We estimated that 26 participants would be required when setting a type I error of 5% and a type II error of 20%. To account for the 5% sample loss, we recruited 28 participants. (n=14 in the FOI group, n=14 in the VAFI group). 

## Results

A total of 28 participants were enrolled in the study, and none were excluded from participation. There was no significant difference in the participants’ characteristics in each group (Table 1).

**Table 1:** Participants’ characteristics (n=28) FOI: fiberoptic intubation; VAFI: video-assisted fiberoptic intubation; IQR: interquartile range; SD: standard deviation

	FOI (n=14)	VAFI (n=14)	p-value
Age, year, median (IQR)	27 (26-33)	29 (25-48)	0.68
Sex, female, n (%)	12 (85.7)	11 (78.6)	1.00
Post-graduate year, year, mean (SD)	5 (3-7)	7.5 (3-19)	0.45

Regarding the primary endpoint, there was no significant difference in the time until the completion of tracheal tube placement, with a median (IQR) time of 55.0 (32, 100) seconds (FOI group) and 42.5 (27, 67) seconds (VAFI group) (p = 0.22). Regarding the secondary outcomes, there was no statistically significant difference between the two groups in any of these outcomes (Table 2).

**Table 2:** Secondary outcomes of time until the tracheal tube passes through the vocal cords, the number of intubation attempts, and the first-success rate (n=28) FOI: fiberoptic intubation; VAFI: video-assisted fiberoptic intubation; IQR: interquartile range; SD: standard deviation

	FOI (n=14)	VAFI (n=14)	p-value
Time, second, median (IQR)	42.5 (21, 83)	37 (18, 57)	0.42
The number of intubations, mean (SD)	1.21 (0.58)	1.35 (0.74)	0.58
First attempt success, n (%)	10 (71.4)	10 (71.4)	1.00

## Discussion

This randomized, controlled, simulation-based study compared the efficacy of VAFI with FOI in a high-fidelity pediatric simulator by healthcare providers with no prior experience of tracheal intubation. We found no statistically significant difference in the time of intubation, the number of intubation attempts, or the first-success rate with the use of VAFI.

In pediatric anesthesia, the smaller size and anatomy of the pediatric airway, as well as the high oxygen consumption, can make securing the airway more challenging than in adult patients. This increased difficulty can lead to an increased risk of hypoxia, which is a contributing factor to perioperative respiratory adverse events in pediatric patients [9]. Therefore, establishing a faster and safer intubation technique is crucial to reducing the risk of complications.

FOI can be useful for securing the airway in pediatric patients with challenging airways, such as those with congenital abnormalities or other anatomical issues. In such cases, FOI may be the only option for successfully intubating patients [10, 11]. Therefore, pediatric anesthesiologists need to acquire proficiency in this technique. However, the infrequent use of FOI in actual patients can make it difficult for anesthesiologists to acquire and maintain their skills. A study has shown a negative correlation between the number of fiberoptic procedures performed and the rate of complications [12]. Furthermore, using FOI by novice trainees is more time-consuming, with a median intubation time of 117 s compared to 42 s for experts, which will contribute to more frequent complications [13]. The infrequent use of FOI and the potential for increased complications with limited experience highlight the importance of examining more effective methods for performing FOI to reduce the risk of complications. Based on our clinical practice, we estimated that the VAFI could make fiber optic intubation easier, especially for novice anesthesiologists. However, our study did not demonstrate that VAFI is more effective in facilitating novice healthcare providers to perform intubation compared to FOI.

Several factors might have influenced our results. This study only focused on the normal anatomical features, which may have affected our results. Previous studies have reported conflicting findings regarding the efficacy of VAFI compared to video laryngoscopy [7,14]. However, these studies mainly included patients with Cormack-Lehane grade 1 or 2, making the effectiveness of VAFI for patients with difficult airways unclear. Notably, in a difficult cadaver airway model, the success rate of tracheal intubation by VAFI was found to be higher than in video laryngoscopy [8]. Therefore, the appropriate elective patients with difficult airways might benefit from VAFI in adult fields.

Several case reports indicated the usefulness of VAFI for pediatric patients with certain congenital syndromes known for difficult airways [10]. Thus, a further study evaluating the effectiveness of VAFI for pediatric patients with difficult airways is warranted. Additionally, this study did not simulate factors that could disturb a view of FOI during the procedure, such as secretion in the oropharyngeal space. The blurred view of the flexible bronchoscope could make it challenging to locate the site, whereas VAFI allows for navigation of the flexible bronchoscope using video laryngoscopy. Furthermore, VAFI can be a valuable tool for intubation when patients have spontaneous breathing or a reduction in airway tone as a result of sedation because it allows more space to manipulate a bronchoscope. Hence, this study could not demonstrate the potential benefits of VAFI over FOI.

This study has several important limitations. First, the study results may not apply to novice anesthesiologists because all the participants were registered nurses. Second, this study was conducted using a pediatric high-fidelity simulator and not actual patients. Finally, as mentioned above, we could not reflect difficult situations such as copious secretions, which could blur the vision of flexible bronchoscopes and video laryngoscopy.

## Conclusions

In conclusion, this study could not show the superiority of VAFI over conventional FOI in a high-fidelity pediatric simulator for healthcare providers without prior tracheal intubation experience. Several factors influence the results, such as the simulator’s normal anatomy and the absence of copious secretions. We consider VAFI to be beneficial in cases where difficult airway management is required or when disturbances such as saliva are present.

Therefore, further investigations that focus on the efficacy of VAFI for difficult airways are recommended to explore the efficacy of VAFI for pediatric patients with difficult airways.
